# Renal arteriovenous fistula after retrograde ureteroscopic lithotripsy for the lower ureteral stones: a rare case report

**DOI:** 10.1186/s12894-020-00688-1

**Published:** 2020-08-15

**Authors:** Wan-Zhang Liu, Ting Huang, Li Fang, Yue Cheng

**Affiliations:** Department of Urology, NingBo First Hospital, NingBo Hospital of ZheJiang University, #59 Liuting Street, NingBo City, 315000 Zhejiang Province China

**Keywords:** Renal arteriovenous fistula, Severe bleeding, Computed tomography angiography, Digital subtraction angiography, Case report

## Abstract

**Background:**

Renal arteriovenous fistula was rarely reported in retrograde endoscopic procedure. Up to now, there is still an absence of report on the formation of renal arteriovenous fistula after semi-rigid ureteroscopic lithotripsy for lower ureteral stones.

**Case presentation:**

An 83-year-old man was admitted to our hospital complaining about intermittent left flank pain that had persisted for 1 week. He suffered medium hypertension and nephrolithiasis treated with left open ureterolithotomy and two ureteroscopic lithotripsies. Non-contrast abdominal CT scan revealed two left lower ureteral stones diametered 8 mm and 7 mm respectively with mild hydronephrosis. A retrograde semi-rigid ureteroscopic lithotripsy was performed to remove the stones, after which two Double-J stents were placed for the ureteral stricture. Due to the continuous gross hematuria and hemoglobin droppings 2 days after operation, a variety of conservative therapies, including blood transfusion and bed rest, were adopted. Then, the patient was discharged with a stable hemoglobin. However, he presented himself to our emergency department with aggravating left flank pain and severe gross hematuria as little as 2 days later. Emergent digital subtraction angiography was conducted to reveal an arteriovenous fistula in the left kidney, which was embolized with two platinum coils to stop the bleeding. His hematuria was resolved in 3 days, and two Double-J stents were removed in 4 weeks. The patient was followed up for 1 year, during which no hematuria or flank pain recurred.

**Conclusion:**

This is the first case report on the formation of renal arteriovenous fistula after semi-rigid ureteroscopic lithotripsy. In this case, elevated intrapelvic pressure, historical surgery and hydronephrosis might be associated with the primary risk of the complication.

## Background

At present, retrograde semi-rigid URSL has been commonly applied in the observation and treatment of ureteral diseases. It is widely known that, the incidence of postoperative complications is low, mostly manifested as Grade I and II, for example, renal colic, infection, ureteral injury and hematuria [1]. The formation of AVF after retrograde procedure was rarely seen, as a result of which there have been few reports on the retrograde intrarenal lithotripsy for renal stones over the past two decades [2]. Therefore, a patient exhibiting an AVF after semi-rigid URSL in the treatment of the lower ureteral stones is reported in this study, and the relevant literature is reviewed.

## Case presentation

An 83-year-old man was referred to our clinic in May 2019 for the complaint about intermittent left flank pain that had persisted for 1 week. Despite the denial of fevers, dysuria, hematuria, frequency or urgency, he admitted to visiting local clinics and receiving medical therapies. Besides, this patient had medium hypertension and nephrolithiasis treated with left open ureterolithotomy and two URSLs.

**Fig. 1:**
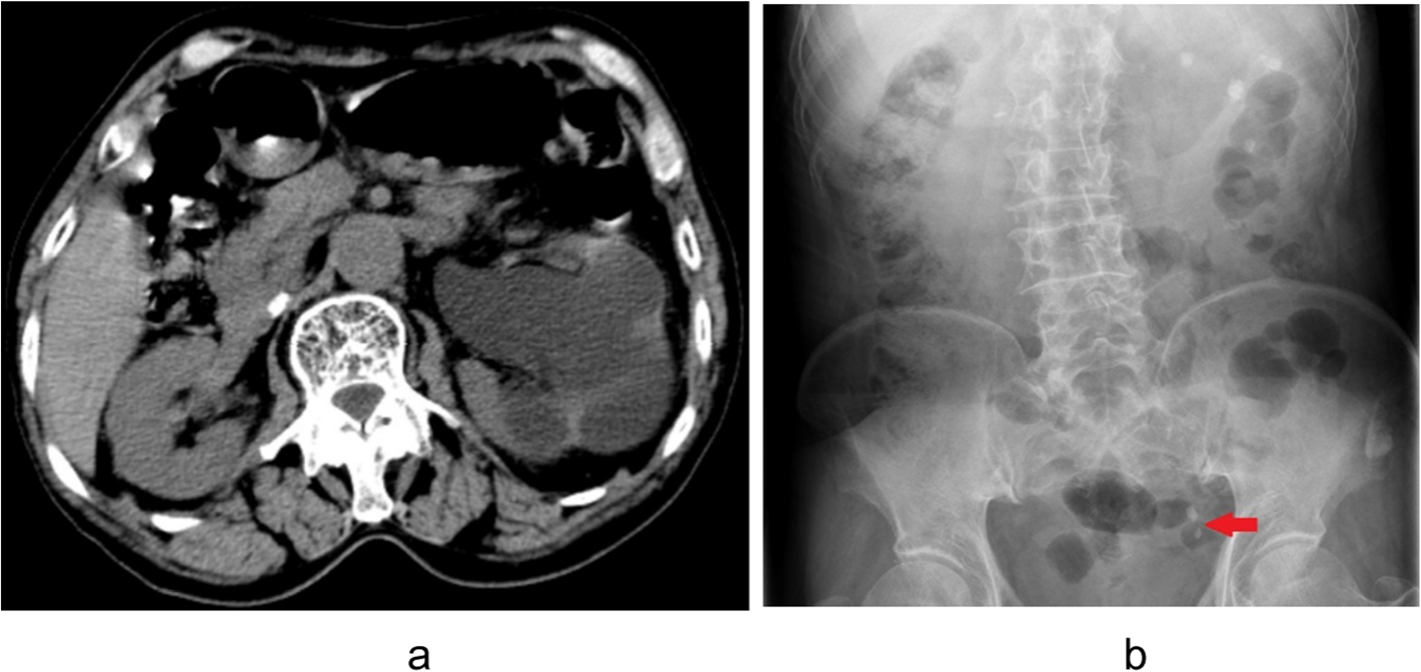
**a** CT revealed mild hydronephrosis in the left kidney. **b** KUB exhibited two lower ureteral stones

Though routine urinalysis revealed 40 WBC/ul and 48 RBC/ul, the result of urine culture was negative. As revealed by non-contrast abdominal CT scan, there were two left lower ureteral stones diametered 8 mm and 7 mm respectively with mild hydronephrosis, as shown in Fig. 1a. In addition, kidney, ureter, bladder (KUB) plain film indicated two lower ureteral stones, as show in Fig. 1b. In spite of this, both preoperative physical examination and laboratory study revealed no abnormality. After the identification of left ureteral stones, semi-rigid URSL with holmium laser was adopted. The surgery was performed by a surgeon with more than 5 years of specialist experience.

As preparation for the operation, the general anesthesia was conducted for 47 min. With the patient was left in the lithotomy position, a 6/7.5-Fr semi-rigid ureteroscope (Storz, Germany) with 220 μm holmium laser was applied to smash the stones. Upon close examination, an ureteral stricture was found beneath the stones, and the semi-rigid ureteroscope passed it carefully with the guidance of a guide wire. For improved visibility, a hand-pump irrigation was carried out by an assistant. The process of smashing stone continued until the visible fragments were ≤ 3 mm in diameter, with most of them extracted into the bladder using a basket. Finally, two 5Fr Double-J stents were placed due to the ureteral stricture and an urethral catheter was indwelled.

**Fig. 2:**
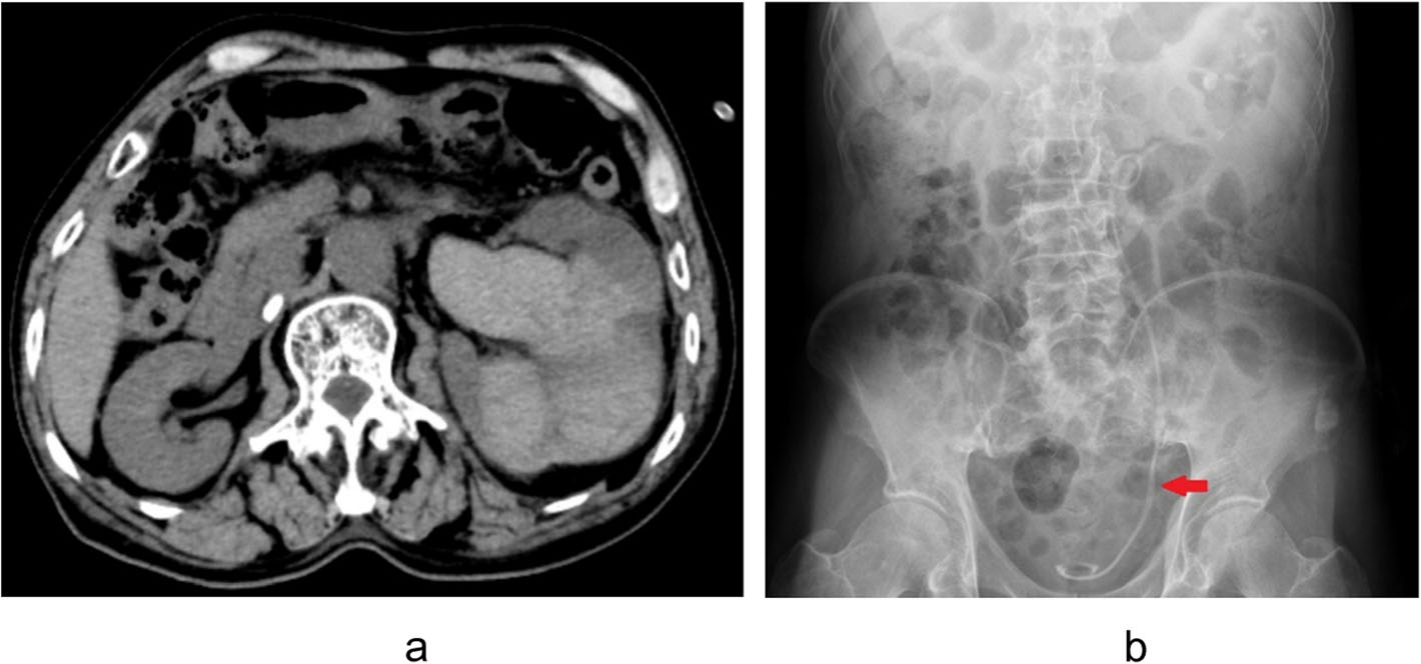
**a** CT revealed mild left hydronephrosis with blood clots. **b** KUB showed two ureteral stents in the left ureter

The patient displayed slight left flank pain and continuous hematuria after the surgery, which was considered as a complication of Double-J stents. Normal blood tests indicated severe droppings of hemoglobin from 103 g/dL to 61 g/dL 2 days after operation. Low dose abdominal CT-scan revealed mild left hydronephrosis with blood clots, while no remains of stones were observed in the ureter, as shown in Fig. 2a. KUB showed two ureteral stents in the left urinary tract, as shown in Fig. 2b. Due to the continuous gross hematuria and droppings of hemoglobin, various conservative therapies, including blood transfusion (600 ml of red blood cells in total), antibiotics (sulbenicillin) and bed rest, were carried out. After 10 days of observation in hospital, the patient was discharged with a slight hematuria and stable hemoglobin level (84 g/dL). Over the course of observation, creatinine and other laboratory tests invariably showed compliance with the specified limits. Moreover, the patient was instructed to remove his two Double-J stents in 4 weeks.

**Fig. 3:**
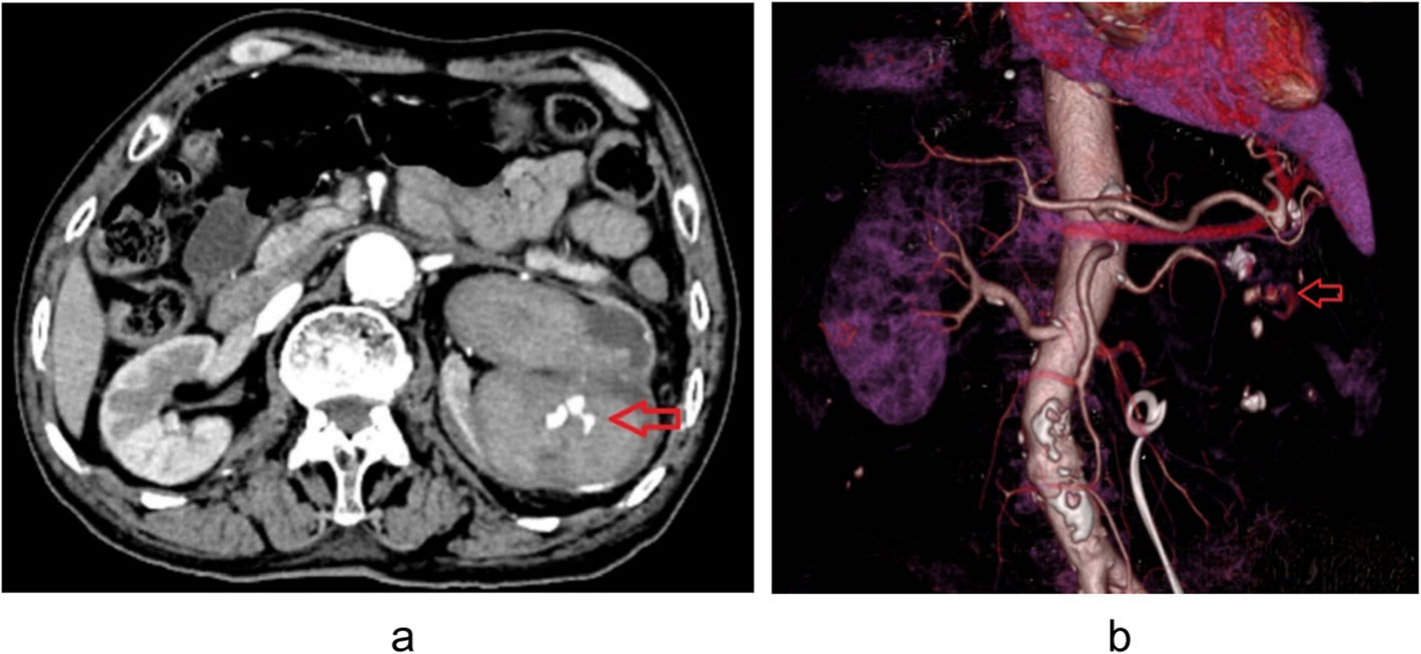
**a** multi-detector computed tomography angiography showed mild hydronephrosis with blood clots and AVF in the left kidney. **b** three-dimensional construction of renal arteries found an AVF

**Fig. 4:**
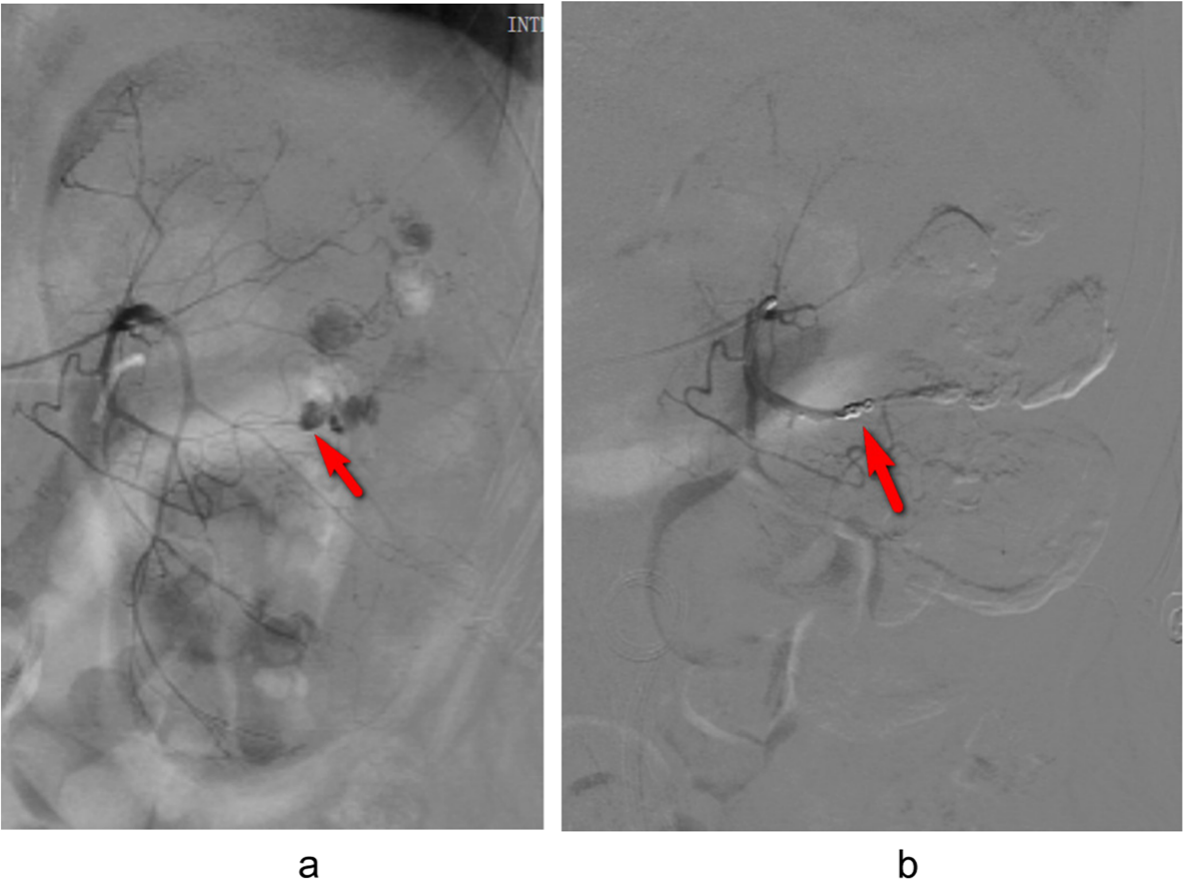
**a** digital subtraction angiography revealed an AVF in the left kidney. **b** the AVF was embolized with two platinum coils

Two days later, he presented himself to our emergency department with aggravating left flank pain and severe gross hematuria. Four thousand seven hundred eighty-three RBC/ul and 364WBC/ul were detected through urinalysis. Blood test revealed low hemoglobin level at 54 g/dL. According to the multi-detector computed tomography angiography and three-dimensional construction of renal arteries, there was an AVF and contrast leakage into calices in the left kidney, as shown in Fig. 3a and Fig. 3b. Then, the findings were confirmed by emergent digital subtraction angiography, as shown in Fig. 4a. Additionally, the AVF was embolized with two platinum coils to stop bleeding, as shown in Fig. 4b. In the meantime, blood transfusion was conducted until the hemoglobin levels reached 70 g/dL. Hematuria was resolved in 3 days and two Double-J stents were removed 1 month later. During the 12-month follow-up, no hematuria or flank pain recurred.

## Discussion and conclusion

Despite renal hemorrhage as a common complication caused by urological endoscopic procedure, it was mostly studied in percutaneous operations, such as PCNL. It might be manifested as pseudoaneurysm, AVF, bleeding spot and combined [3]. Since the kidney is classed as a vascular organ, bleeding might occur as a result of the damage caused to the renal substances, like the introduction of needle, dilatation of tract, fragmentation of stones and elevated intrarenal pressure. In spite of conservative therapies, further digital subtraction angiography and embolization are required to stop bleeding for those with continuous gross hematuria and a severe hemorrhage (hemoglobin dropping>30 g/dL) [4].

Different from PCNL, retrograde ureteroscopic surgery is completely performed through the urinary tract, so as to avoid the potential substantial damage caused to the kidney. Thus, the incidence of renal hemorrhage following retrograde procedures was extremely low (0.15–0.4%) [5–7], and it was mostly manifested as subcapsular and perinephric hematoma. It was believed that the damage caused to pelvicalyceal system by guide wire and the elevated intrarenal pressure leading to the rupture of renal parenchyma might be attributed to bleeding [7]. Kozminski [8] suggested that renal hematoma is associated with such factors as female gender, preoperative hypertension, preoperative ureteral stenting, intraoperative ureteral sheath use, and postoperative ureteral stenting. Tiplitsky [2] presented a case of intrarenal arteriovenous fistula after the flexible ureteroscopic extraction of stone with holmium laser lithotripsy, which led to the finding that the damage caused to interlobar arteries by holmium laser was the most likely root cause. As we known, there is still no report on the formation of AVF following semi-rigid ureteroscopic lithotripsy for the lower ureteral stones.

In our case, the elevated intrapelvic pressure during the process of smashing stones is supposed to be the leading cause of AVF and bleeding. On the one hand, the ureteral stricture beneath the stones could reduce not only the gap between the ureteral wall and the ureteroscope, but also the outflow of irrigative fluid as a consequence. Secondly, a hand-pump irrigation, performed by an assistant, provided a high irrigative pressure to ensure the sufficient visibility. In the meantime, however, it caused the intrarenal pressure to increase. But we were not able to measure the exact pressure during the surgery. On the other hand, a long-term hydronephrosis and previous surgery histories could have a detrimental effect on the substantial of kidney, thus leading to the potential formation of AVF.

It marks the first case in relation to the formation of AVF after semi-rigid ureteroscopic procedure, but the exact physiopathology remains unclear. To our knowledge, the elevated intrapelvic pressure that might lead to the rupture of renal parenchyma was mainly responsible for the complication. For the patients with a ureteral stricture beneath the stones, the double-J stent should be placed 2-4 weeks in advance to facilitate the passage of endoscope and the outflow of irrigation while the smashing of stones proceeds. Although the usage of double ipsilateral ureteral stents achieved a higher success rate than single stent in the treatment ureteral strictures after an endoureterotomy [9], the efficiency of two stents placement without an endourological technique in this case is still lack of evidence. Finally, surgeons must be aware of the outflow of irrigative fluid, but not always a good visibility during the smashing of stones.

## Data Availability

Not applicable.

## Abbreviations

AVF: Arteriovenous fistula
URSL: Ureteroscopic lithotripsy
WBC: White blood cell
RBC: Red blood cell
CT: Computed tomography
PCNL: Percutaneous nephrolithotomy
